# Doublecortin-immunoreactive neurons in the piriform cortex are sensitive to the long lasting effects of early life stress

**DOI:** 10.3389/fnins.2024.1446912

**Published:** 2024-09-16

**Authors:** María Abellán-Álvaro, Anna Teruel-Sanchis, Maria Francisca Madeira, Enrique Lanuza, Mónica Santos, Carmen Agustín-Pavón

**Affiliations:** ^1^Unitat Mixta d’Investigació en Neuroanatomia Funcional, Departament de Biologia Cel⋅lular, Biologia Funcional i Antropologia Física, Universitat de València, València, Spain; ^2^CNC-UC—Center for Neuroscience and Cell Biology, University of Coimbra, Coimbra, Portugal; ^3^Doctoral Programme in Experimental Biology and Biomedicine (PDBEB), Institute for Interdisciplinary Research, University of Coimbra, Coimbra, Portugal; ^4^CIBB—Centre for Innovative Biomedicine and Biotechnology, University of Coimbra, Coimbra, Portugal

**Keywords:** maternal separation, MeCP2, neurogenesis, olfactory cortex, olfactory bulbs, sex differences

## Abstract

The olfactory system is a niche of continuous structural plasticity, holding postnatal proliferative neurogenesis in the olfactory bulbs and a population of immature neurons in the piriform cortex. These neurons in the piriform cortex are generated during embryonic development, retain the expression of immaturity markers such as doublecortin, and slowly mature and integrate into the olfactory circuit as the animal ages. To study how early life experiences affect this population of cortical immature neurons, we submitted mice of the C57/Bl6J strain to a protocol of maternal separation for 3 h per day from postnatal day 3 to postnatal day 21. Control mice were continuously with their mothers. After weaning, mice were undisturbed until 6 weeks of age, when they were weighted and tested in the elevated plus-maze, a standard test for anxiety-like behavior, to check for phenotypical effects. Mice were then perfused, and their brains processed for the immunofluorescent detection of doublecortin and the endogenous proliferation marker Ki67. We found that maternal separation induced a significant increase in the body weight of males, but not females. Further, maternally separated mice displayed increased exploratory-like behavior (i.e., head dipping, velocity and total distance traveled in the elevated plus maze), but no significant differences in anxiety-like behavior or corticosterone levels after behavioral testing. Finally, we observed a significant increase in the number of complex doublecortin neurons in the piriform cortex, but not in the olfactory bulbs, of mice submitted to maternal separation. Interestingly, most doublecortin neurons in the piriform cortex, but not the olfactory bulb, express the epigenetic reader MeCP2. In summary, mild early life stress results, during adolescence, in a male-specific increase in body weight, alteration of the exploratory behaviors, and an increase in doublecortin neurons in the piriform cortex, suggesting an alteration in their maturation process.

## 1 Introduction

The olfactory system is one of the few niches hosting postnatal neurogenesis in the brain of many mammalian species (Paredes et al., 2016). Neural stem cells are born and proliferate in the ventricular-subventricular zone (V-SVZ), and derived young neurons migrate through the rostral migratory stream to reach the olfactory bulb (OB), where they mature mostly as granular interneurons (Doetsch et al., 1999). This form of postnatal neurogenesis is highly sensitive to environmental influences, such as olfactory stimulation (Shapiro et al., 2007) or deprivation (Račeková et al., 2009; Sawada et al., 2011), and to physiological changes (Chaker et al., 2023).

In addition, protracted maturation of embryonic-born neurons takes place in the olfactory (piriform, Pir) cortex. Immature neurons in layer II of the Pir are generated around embryonic day 14–15 in mice (Rubio et al., 2016), express the markers PSA-NCAM and doublecortin (DCX) and slowly mature and integrate into the circuit (Rotheneichner et al., 2018). In contrast to immature neurons in the OB, which are prominent in rodent species, immature neurons might be widespread in layer II of the neocortex in mammalian species with larger brains (La Rosa et al., 2020) and even humans (Coviello et al., 2022). Thus, cortical DCX cells constitute an interesting target that might be relevant for neurological diseases. In this context, we previously found that this population is increased in a mouse model of Rett syndrome (Martínez-Rodríguez et al., 2019). In addition, they are sensitive to olfactory bulbectomy (Gómez-Climent et al., 2011) and chronic stress (Nacher et al., 2004), but studies about the effects of early life experiences on them are scarce.

The influence of early life stress (ELS) has been extensively studied in animal models and humans, due to their long-lasting effects on the development and function of the nervous system, leading to increase in the vulnerability to develop psychiatric disorders. Thus, ELS leads to epigenetic changes in the central nervous system, causing long-term effects in neuroendocrine and behavioral responses of individuals (Thumfart et al., 2022). Among early life experiences that lead to persistent epigenetic changes, maternal care is key in shaping stress reactivity, social behavior, and cognitive functions (Cameron and Garcia, 2013).

Epigenetic changes are engraved in our DNA in form, for example, of specific methylation patterns, that are read by epigenetic regulators such as methyl-CpG-binding protein 2 (MeCP2) (Murgatroyd et al., 2009). We recently showed that a mild maternal separation-induced ELS, for 3 hours per day from postpartum day 2 until weaning, lead to an increase of the population of immature neurons of the Pir, but no in the OB, in *Mecp2*-null males, model of Rett syndrome, and their WT controls (Torres-Pérez et al., 2022). Further, we found a specific increase in weight in maternally separated WT male mice, as compared with those under continuous maternal care. However, in that study we analysed only male experimental subjects, but ELS interacts with sex, revealing sex-specific differences in response to early stressors which may predispose individuals to various psychiatric conditions (Knox et al., 2021). Moreover, dams of the analysed litters were *Mecp2*-het, that display impoverished maternal care (Krishnan et al., 2017), which might have influenced the observed results. Thus, in the present study, we aimed to extend our findings to a commonly used mouse strain, the C57/Bl6J strain, to discard the possible effects of abnormal maternal care, and analyse both males and females.

## 2 Materials and methods

### 2.1 Animals

For the study about the effects of MS, we paired 8 young adult female mice with 8 young adult male mice (*Mus musculus*) from the C57/Bl6J strain (Janvier, Le Genest Saint-Isle, France). The stud males were separated from the dams once pregnancy was confirmed, and dams were randomly assigned to Continuous Care (CC, *n* = 4 dams) or Maternal Separation (MS, *n* = 4 dams). From these dams, we obtained 26 female mice (CC, *n* = 12; MS, *n* = 14) and 21 male mice (CC, *n* = 13; MS, *n* = 8). In addition, we used 2 male and 2 female mice (6–8 weeks old) control mice for immunofluorescent detection of DCX and MeCP2.

All animals were housed in cages with water and food available *ad libitum* with 12 h light:dark non-inverted cycle at 22–24°C. All procedures were carried out between 9:00 a.m. and 14:00 p.m., at light phase. Mice were treated according to the guidelines of the European Union Council Directive of June 3rd, 2010 (6106/1/10 REV1). All the experimental procedures were approved by the Committee of Ethics on Animal Experimentation of the University of Valencia.

### 2.2 Maternal separation protocol

Pups from the MS litters were separated from their mothers for 3 h per day, daily (10:00 a.m. to 13:00 p.m.), between postpartum day (PPD) 2 to PPD21. The whole litter was kept in a separate room in a cage with clean bedding, and warmed with a heating blanket. After separation, pups were gently returned to their home cage. Pups from the CC litters were facility-reared, i.e., maintained in the home cage with their mother until weaning at PPD21. At weaning, mice were housed with their same-sex siblings (*n* = 3–5 per cage) and then tested at 6 weeks of age, a time point comparable with human adolescence (Brust et al., 2015), when postnatal neurogenesis is still at its peak (Arellano et al., 2024). Change of bedding was performed weekly by the facility personnel in all groups of mice.

### 2.3 Behavioral testing and analysis

For behavioral testing we used the elevated plus maze apparatus (EPM), that allows measuring both exploratory and anxiety-like responses in rodents (Pellow et al., 1985), as it challenges the mouse preference for their innate exploratory behavior versus fear of heights/unknown (Barnett and Smart, 1975). The EPM was a gray Plexiglas apparatus consisting of four 30-cm-long and 5-cm-wide arms (two open and two enclosed by 15.25 cm high walls). Each arm of the maze was attached to 40 cm long metal legs. At 6 weeks of age, all mice from the CC and MS groups were transported in their home cages (same sex animals) to the testing room, with the same lighting conditions as the home room. Mice were gently left in the middle of the EPM and their behavior was video recorded for 5 min. The EPM apparatus was thoroughly cleaned with 70% ethanol and allowed to dry between each subtect.

Behavioral tracking was analysed with DeepLabCut (version 2.2.0.6, Mathis et al., 2018). We created a network to track the points of interest: nose, right ear, left ear, head, spine 1, spine 2, base tail (Supplementary Figure 1). We extracted 20 frames from 34 randomly selected tracking videos (30 fps), using k-means clustering to represent behavioral diversity. Then, we trained a network (ResNet-50) (Insafutdinov et al., 2016) for 160 000 iterations within the computer cluster of the Bioinformatics and Biostatistics Unit in Principe Felipe Research Center (CIPF, Valencia, Spain) achieving a train error of 4.18 pixels and test error of 5.33 pixels. We then used a p-cutoff of 0.9 for X, Y coordinates refinement for future analysis. We used this network to analyse videos from same experimental settings.

Subsequent data was then processed using self-written Python code. We analysed the time spent in the closed and open arms, distance traveled, head-dipping events and number of entries in the open and closed arms. Exploration was considered positive when the center of mass was inside the region of interest, defined by top-left and bottom-right coordinates from each EPM arm. Head dipping was considered when the snout was outside the open arms (Supplementary Figure 1). These results were validated by a researcher blind to the experimental groups using the SMART 3.0 video tracking system (Panlab, Cornellà, Barcelona, Spain) (Supplementary Figure 2).

### 2.4 Sacrifice and histology

Ninety minutes after the EPM, mice received an overdose of sodium pentobarbital (intraperitoneal, 100 mg/kg, Eutanax, Laboratories Normon S.A. Madrid, Spain). Blood was withdrawn from the aorta, kept in heparinized 1.5 ml tubes and centrifuged for further analysis. Plasma was stored at −20 C until use. Then, mice were transcardially perfused with saline solution (0.9%) followed by 4% paraformaldehyde diluted in PB (0.1M, pH 7.6). Brains were carefully removed from the skulls, post-fixed for 4 hours in the same fixative, and cryoprotected until they sank in 30% sucrose in 0.1 M PB at 4°C. The tissue was sliced into 40 μm coronal sections using a freezing microtome (Leica) and collected in six parallel series.

### 2.5 ELISA

We measured corticosterone levels using a commercially available competitive ELISA kit for the measurement of corticosterone (Ab108821, Abcam), according to the protocol provided by the company. Briefly, plasma samples were diluted 1:100 in sample diluent, pipetted onto an ELISA plate precoated with an antibody against corticosterone, and incubated with a biotinylated corticosterone protein. The Streptavidin-Peroxidase conjugate was added next, followed by the chromogen substrate 3,3′, 5,5′-Tetramethylbenzidine (TMB). All reagents were provided by the kit. After halting the reaction, absorbance was read in a Spectramax plus 384 microplate spectrophotometer (Molecular Devices, LLC, USA) at 450 nm. The standard curve was plotted using a four-parameter logistic curve-fit.

### 2.6 Immunofluorescence and confocal imaging

We carried out double immunofluorescent labeling for DCX and Ki67, an endogenous marker of proliferation, in one of the series of CC and MS subjects, and DCX and MeCP2 in one series of control subjects. To block the endogenous fluorescence of the tissue the slices were incubated with 1% sodium borohydride (Fluka) in 0.05 M TBS for 30 min at room temperature (RT). Next, for DCX-MeCP2 sections, we performed antigen retrieval by incubating the sections for 30 minutes in citrate buffer (0.01 M, pH 6) at 80°C. Once the tissue reached room temperature, it was washed again three times with TBS for 5 minutes each. Sections were then incubated in a blocking solution containing 3% normal donkey (NDS) and goat (NGS) serum in TBS-Tx 0.3% for 1 h at RT. Afterward, each series was incubated for 48 h at 4°C with a mixture of primary antibodies containing guinea pig anti-DCX (1:4000, AB2253, Sigma-Aldrich) and rabbit anti-Ki67 (1:5000, SAB5700770, Sigma-Aldrich) or rabbit anti-DCX (1:000, AB18723, Abcam) and mouse anti-MeCP2 (1:1000, MA5-33096, Invitrogen) diluted 1:1000 in TBS-Tx 0.3%/NDS and NGS 2%. Next, brain slices were incubated for 90 min at RT with appropriate secondary antibodies Rhodamine Red™-X Donkey anti-Rabbit (1:200, 711-295-152, Jackson ImmunoResearch), Alexa Fluor^®^ 488 goat Anti-guinea pig (1:200, A-11073, Invitrogen) or Alexa Fluor-488 Dk α Ms (1:200, A21202, Invitrogen) in TBS-Tx0.3% with 2% NDS and NGS. Finally, sections were incubated with 600 nM DAPI (4’,6’-diamidino-2-phenylindole) in TBS for 1 min at room temperature and mounted onto gelatinized slides and cover-slipped with fluorescence mounting medium (Fluoromount, Merck).

For DCX-Ki67 samples, we obtained fluorescence images of Pir (magnification 63X) and OB (magnification 10X) using laser scanning confocal microscope (FV1000, Leica, SPE, Leica Microsystems, Wetzlar, Germany), obtaining Z project stacks of confocal images with 1μm Z-step size. All the photomicrographs were taken in previously selected Bregma levels, according to Paxinos and Franklin mouse brain atlas (2012). For the OB, we analysed 3 coronal sections from Bregma + 3.2 to + 3 and made a picture centered at the granular cell layer. For the Pir, we analysed 3 coronal sections from Bregma from 0 to −2, taking at least 4 pictures centered at layer II for each hemisphere, where the DCX cells are located. All pictures were taken by the same person using the same conditions in the confocal microscope.

For DCX-MeCP2 analysis, we found the DCX neurons and selected the focal plane in which the nucleus was most visible using the DAPI staining. A single plane confocal stack was taken and co-expression of DCX and MeCP2 was determined by visual inspection with the channels tool of Fiji.

### 2.7 Image analysis

Quantification of DCX-ir cells was performed in Fiji (NIH). Due to the high-density cells and fibers in the OB, we analysed DCX and Ki67 expression by means of fluorescence intensity (Torres-Pérez et al., 2022). First, images were split into the RGB channels. The relevant channel for the marker of interest was selected, and the optical density (OD) of the inverted image was calculated as Log10 = Max Intensity/Mean Intensity in each channel. For the Pir, DCX cells were manually counted in each picture (area: 175 μm^2^). To classify Pir neurons as tangled or complex, we followed a previously described method based on feret diameter (Rubio et al., 2016; Coviello et al., 2021), so neurons with a feret diameter smaller than 10 μm were classified as tangled. The average density of neurons was calculated by number of DCX-ir/photo.

### 2.8 Statistical analysis

Data were analysed using RStudio (2021.09.2) and SPSS 26.0. Firstly, we checked for normality (Shapiro–Wilk test), homoscedasticity (Levene’s test) and sphericity (Mauchly’s test). Behavioral and histological data were analysed with two-way ANOVA with group (CC or MS) and sex (female or male) as between-subject factors, followed by *post hoc* with the Bonferroni correction for multiple tests. When data did not follow the assumptions of the ANOVA, we used the non-parametric Mann-Whitney test. Significance was set at *p* < 0.05 for individual factors, and we carried out *post hoc* pairwise comparisons when *p* < 0.1 for the interaction between factors.

## 3 Results

### 3.1 Maternal separation has long lasting effects on weight and exploratory activity

**FIGURE 1:**
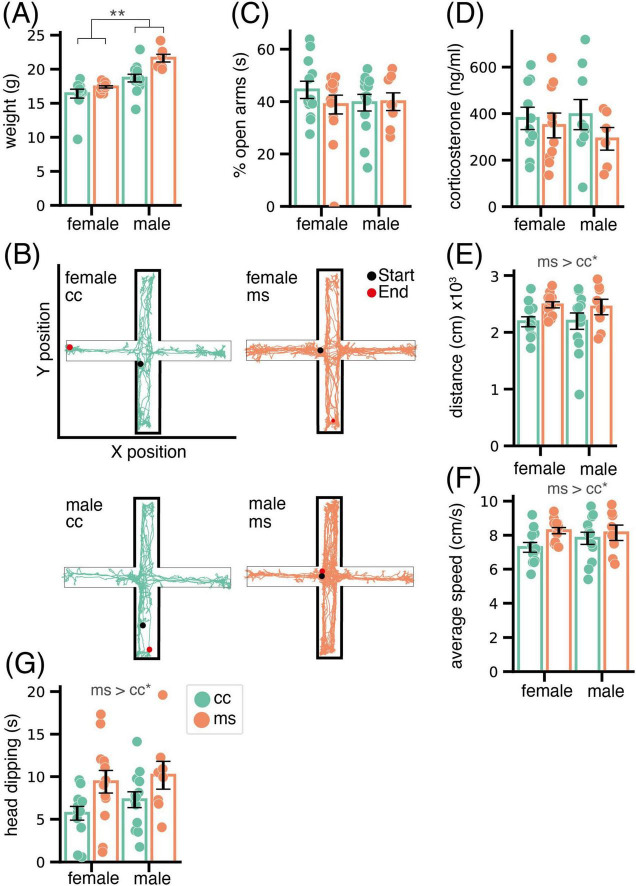
Phenotypic consequences of MS in adolescent mice. **(A)** Bar chart showing weight at 6 weeks of age of female and male mice. Statistical analysis shows, as expected, significant weight differences between males and females, and a further effect of MS in increasing the weight of males. **(B)** Representative behavioral tracking of mice in the EPM. Neither percentage of time spent in the open arms of the EPM **(C)** or corticosterone plasma levels after this test **(D)** reveal significant differences due to group or sex, suggesting no effect of MS in the anxiety-like profile. By contrast, distance traveled **(E)**, average speed **(F)** and head dipping **(G)** were significantly increased in adolescent mice of both sexes that were subjected to ELS. Data are shown as Mean ± SEM. * *p* < 0.05; ** *p* < 0.01.

Animals were weighed before the EPM test, and the ANOVA revealed an expected significant effect in weight of the factor sex (F_1,41_ = 37.23, *p* < 0.001), with males being heavier than females. We also found a significant effect of group (F_1,41_ = 13.54, *p* < 0.001), with MS inducing an increase in weight (Figure 1A), and an interaction of both factors (F_1,41_ = 3.32, *p* = 0.076). *Post hoc* pairwise comparisons revealed that MS males were significantly heavier than CC animals (*p* < 0.001), but this effect was not significant for the females (*p* = 0.160) (Figure 1A).

Representative cases of behavioral tracking in the EPM of each animal group and sex are shown in Figure 1B. The ANOVA did not reveal any significant effect of the factors group or sex nor their interaction (all *p* > 0.1) in the percentage spent by the animals in open arms, the classical measure associated to anxiety (Figure 1C). Consistent with the lack of differences in anxiety-like behavior of the animals, plasma corticosterone levels measured 90 minutes after the EPM did not reveal significant differences between groups or sexes, and did not correlate with any of the behavioral measures taken (all p > 0.1, Figure 1D).

By contrast, motor and exploratory behavior were significantly affected by MS. Thus, total distance traveled and speed were significantly increased by MS (distance, F_1,42_ = 6.04, *p* = 0.02, Figure 1E; speed F_1,42_ = 4.18, *p* = 0.04, Figure 1F). Further, time spent in head dipping was also increased by MS (F_1,42_ = 7.71, *p* < 0.001, Figure 1G). In all cases, there was no significant effect of sex (*p* > 0.05) or interaction between sex and group (*p* > 0.1).

### 3.2 Maternal separation increases the number of complex doublecortin neurons in the piriform cortex

**FIGURE 2:**
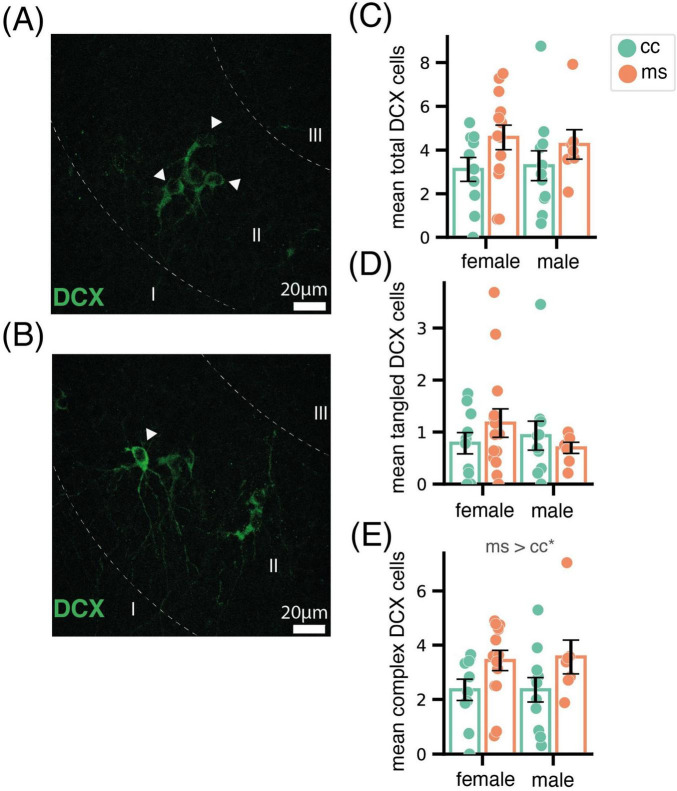
Effect of MS in doublecortin neurons in the Pir. Representative images of the Pir displaying a group of tangled **(A)** and a complex **(B)** immature neurons immunoreactive for DCX. Bar charts showing the mean total **(C)**, tangled **(D)** and complex **(E)** DCX neurons, revealing that MS increased the density of the latter. Data are shown as Mean ± SEM. * *p* < 0.05.

In the Pir, we analysed tangled (Figure 2A), complex (Figure 2B) and the total (sum of both types) DCX-ir neurons. Total DCX-ir cells were not affected by sex (F_1,38_ = 0.01, *p* = 0.91), and only a mildly by group (F_1,38_ = 3.62, *p* = 0.06) (Figure 2C). Similarly, we did not find significant effects of any factor for DCX-ir tangled cells (sex F_1,38_ = 0.38, *p* = 0.54; group F_1,38_ = 0.82, *p* = 0.77, Figure 2D). By contrast, we found a significant increase in complex DCX-ir neurons in mice of both sexes subjected to MS (group F_1,38_ = 6.35, *p* = 0.01; sex F_1,38_ = 0.21, *p* = 0.88; sex x group F_1,38_ = 0.20, *p* = 0.89, Figure 2E). As expected, we did not find significant labeling of Ki67, and none of the DCX-ir cells was co-labeled or close to Ki67-ir nuclei, discarding that the increase in DCX-ir complex neurons was due to proliferative activity.

**FIGURE 3:**
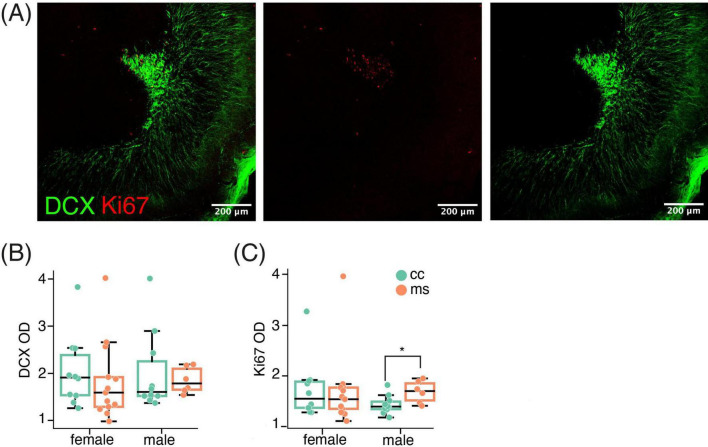
MS did not affect doublecortin in the OB. **(A)** Representative image of the OB displaying both DCX and Ki67 immunoreactive cells. **(B)** MS did not affect DCX expression in the OB, although it increased Ki67 nuclei in males **(C)**. Data are shown as Mean ± SEM. * *p* < 0.05.

Data from the OB were analysed by Mann-Whitney tests to check for the effect of MS in same-sex groups. In the OB, we found Ki67 labeling (Figure 3A), that was not significantly affected by MS in females (*p* = 0.52), but was increased in males (*p* = 0.022, Figure 3C). However, this increase in proliferative nuclei was not translated into an increase in immature neurons, since the analysis did not reveal any significant difference in the density of DCX-ir in the OB in females or males (all *p* > 0.1, Figure 3B).

### 3.3 Most doublecortin neurons in the piriform cortex, but not olfactory bulb, express MeCP2

**FIGURE 4:**
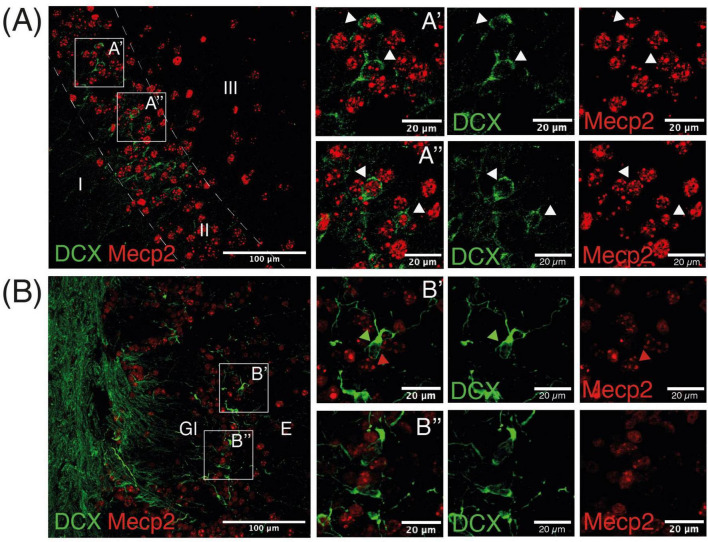
Doublecortin neurons in the Pir express MeCP2. Representative image of the Pir **(A)** and OB **(B)** displaying both DCX cells and MeCP2 immunoreactive nuclei. White arrows in insets in A’ and A” point to DCX neurons expressing MeCP2, green and red arrows in B’ and B” point to DCX somata and MeCP2 nuclei, respectively.

Following the significant effect of MS in Pir but not OB shown by the current study and a previous one using *Mecp2*-null mice (Torres-Pérez et al., 2022), we sought to explore the expression of the epigenetic reader MeCP2 in both populations. We observed that most DCX neurons in the Pir expressed MeCP2 (Figure 4A). Co-labeling in the OB was more difficult to find, where most DCX-ir neurons do not express MeCP2 (Figure 4B). We did not find differences between sexes in this co-expression.

## 4 Discussion

In this study, we found that mice subjected to MS for 3 h per day during infancy display significant phenotypic and neuronal differences when analysed at adolescence. In particular, MS increased weight in a sex-specific way, with males, but not females, being heavier than their controls. MS did not affect anxiety-like behavior in the EPM or corticosterone plasma levels after this test, but increased locomotion and head dipping in both sexes. Furthermore, our results are in agreement with a previous study, showing an increased density of complex DCX-ir, immature neurons in the Pir, which express the epigenetic reader MeCP2, but not in the OB.

Maternal separation is commonly used as a model of ELS in rodents that induces long-term emotional dysregulation (Martín-Sánchez et al., 2022). Many factors, including the duration of the separation, the age at testing and sex of the experimental subjects, the occurrence or not of early weaning, the species (mice or rats) and the strain used lead to specific effects. These effects range from non-significant to an increase or decrease in anxiety-like responses or weight (Tan et al., 2017; Wang et al., 2020; Abellán-Álvaro et al., 2021; Knox et al., 2021; Martín-Sánchez et al., 2022; Torres-Pérez et al., 2022). In our study, MS only increased the weight of adolescent males. By contrast, a study in rats showed that 3 h daily MS increased the weight of infant males and young adult females, with no effect during adolescence (Jaimes-Hoy et al., 2016). Other studies have shown that MS increases the weight of infant MS mice (Bailoo et al., 2014). The weight gain could be attributed to multiple factors, such as the impact of separation on nursing behaviors (Bailoo et al., 2014; Gracia-Rubio et al., 2016) or long lasting neuroendocrine changes (Wu et al., 2014; Jaimes-Hoy et al., 2016).

On the other hand, MS mice of both sexes demonstrated increased locomotion, also observed in previous studies on mice (Tan et al., 2017), and exploratory behavior. These findings might be interpreted as a heightened exploratory drive, as stress-induced hyperactivity or even enhanced impulsivity (Franco et al., 2020). Head dipping is not typically classified as an anxiety-like behavior; rather, an increase in this behavior may indicate a heightened propensity for risk-taking or exploratory behavior (Toledo and Sandi, 2011). In relation to this, ELS, given certain conditions, has been associated with enhanced resilience to stress later in life and improved coping mechanisms when facing stressors (Santarelli et al., 2017). A mild MS paradigm such as the one used in our study might mimic a more naturalistic model of care, conferring resilience to the pups (Connors et al., 2015). Thus, MS might prime the animal’s stress response, leading to engaging in more risk-taking behaviors when facing a challenging situation during adolescence.

By contrast we did not find significant differences the percentage of time spent in the open arms of the EPM. These results are consistent with a meta-analysis showing that MS did not alter behavior in the EPM in mice (Wang et al., 2020), and also with our previous observations in a *Mecp2*-null strain during the first exposure to this apparatus (Abellán-Álvaro et al., 2021; Torres-Pérez et al., 2022). Consistent with lack of differences in anxiety-like responses, corticosterone levels measured after testing were not affected by MS, although we must acknowledge that the timing used might have missed the peak levels of corticosterone, that usually are measured immediately after EPM (Rodgers et al., 1999; Shoji and Miyakawa, 2021).

The main question of this study was to test whether MS would increase DCX-ir in the Pir of males and females of a common strain, as previously found in *Mecp2*-null male mice and their WT controls (Torres-Pérez et al., 2022). As explained above, DCX neurons in the Pir are generated during embryonic development (Rubio et al., 2016) and, as the animal ages, grow from the small, tangled morphology to develop bigger somata and complex dendritic arborization, to finally mature and integrate in the circuit as glutamatergic neurons (Rotheneichner et al., 2018). Thus, the expression of the immaturity marker DCX decreases with age (Ghibaudi et al., 2023). Our results showing that the population of complex DCX cells is the one increased by MS suggest changes in the maturation rate of these neurons, provoking an arrest in their final step of maturation. An alternative explanation is that ELS might accelerate the transition from tangled to complex cells. If that were the case, looking at older ages might reveal a more pronounced drop in DCX neurons. On the contrary, if ELS delays maturation, DCX would be still increased at older ages. Future studies should investigate these possibilities.

It was previously shown that, whereas chronic restrain stress provoked an increase of immature neurons in the Pir of rats, corticosterone treatment decreased their density (Nacher et al., 2004). This difference might be due to the non-physiological increased levels induced by corticosterone treatment, in comparison with the physiological stress response of the whole organism. As noted above, we did not find significant differences on plasma circulating levels of corticosterone after the EPM in our samples, although we cannot discard differences in basal levels between groups.

By contrast to DCX-ir neurons in the Pir, DCX-ir in the OB was not affected by MS in the current study or the previous one (Torres-Pérez et al., 2022). Interestingly, we found that most DCX-ir neurons in the Pir co-express the epigenetic reader MeCP2, whereas this co-expression was scarcely found in DCX-ir neurons in the OB. Since the expression of MeCP2 is known to correlate with the maturation stage of neurons (Shahbazian et al., 2002), the interpretation of our results should take into account that DCX-ir cells in the OB are continuously forming from V-SVZ precursors, whereas DCX-ir neurons in Pir are embryonically generated. Hence, not only DCX-ir neurons of the Pir might have accumulated relevant epigenetic changes due to ELS, which might be absent in newly-generated OB DCX-ir neurons, but also they express relevant epigenetic readers which could influence their behavior. Further, it should be noticed that we did not investigate migration or survival of DCX neurons in the OB, that might be affected by MS.

In all, our study leaves open several interesting questions. For example, what is the role of MeCP2 in protracted maturation in specific neuronal populations in the olfactory system, and what are the implications of this role for neurodevelopmental disorders linked to mutations in *MECP2*? Further, although DCX-ir neurons in cortical layer II have been demonstrated in many mammalian species (La Rosa et al., 2020), their role remains largely elusive. It is possible that they participate in olfactory learning, that has been shown to be affected by ELS (Czarnabay et al., 2019; Pardasani et al., 2023), and even in social behavior, largely dependent on chemosignals in mice (Martinez-Garcia et al., 2009) and influenced by olfactory cues in humans (Endevelt-Shapira et al., 2017). Future studies are warranted to investigate these open questions.

## Data Availability

The raw data supporting the conclusions of this article will be made available by the authors, without undue reservation.
