# Supplementary material for: Doublecortin-immunoreactive neurons in the piriform cortex are sensitive to the long lasting effects of early life stress
Source: Front Neurosci. 2024 Sep 16;18:1446912. doi: 10.3389/fnins.2024.1446912 (PMC11439882; doi:10.3389/fnins.2024.1446912)
Supplement: Supplementary file 1 [file Data_Sheet_1.pdf]

Supplementary Material

Doublecortin-immunoreactive neurons in the piriform cortex are sensitive to the long lasting effects of early life stress

María Abellán-Álvaro[1†], Anna Teruel-Sanchis[1†], Maria Francisca Madeira[2,3], Enrique Lanuza[1], Mónica Santos[2,3], Carmen Agustín-Pavón[1*]

[1] Unitat Mixta d'Investigació en Neuroanatomia Funcional, Departament de Biologia Cel·lular, Biologia Funcional i Antropologia Física, Universitat de València, Spain

[2] CNC-UC – Center for Neuroscience and Cell Biology, University of Coimbra, Portugal

[3] University of Coimbra, Institute for Interdisciplinary Research, Doctoral Programme in Experimental Biology and Biomedicine (PDBEB)

[4] CIBB - Centre for Innovative Biomedicine and Biotechnology, University of Coimbra, Portugal*

[†] These authors contributed equally to this work

Correspondence:

Carmen Agustín-Pavón, PhD

e-mail: pavon@uv.es

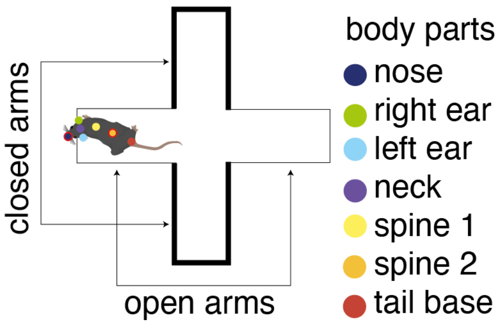

Supplementary Figure 1. Representative layout of the points of interest (nose, right ear, left ear, head, spine 1, spine 2, base tail) for behavioural tracking of the mice in DeepLabCut. Exploration was considered positive when the centre of mass (yellow/red circle) was inside the region of interest. Head dipping was considered positive when the tip of the nose (blur/red circle) was outside the open arms.

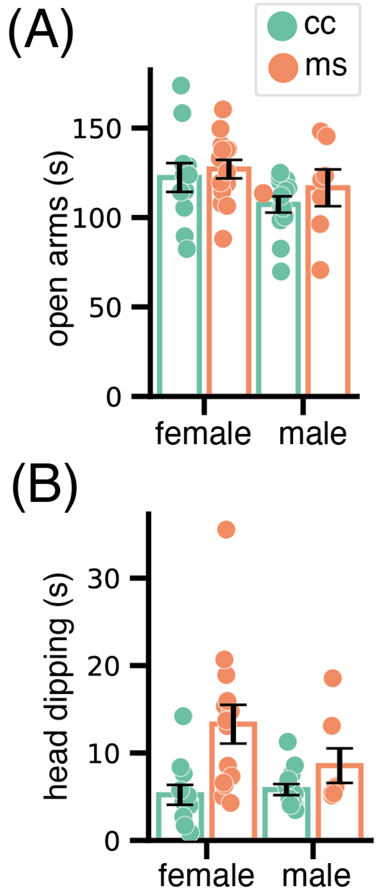

Supplementary Figure 2. Example of behavioural tracking using SMART A) Bar chart showing time in open arms. Statistical analysis shows no significant differences between males and females or effect of MS. B) Bar chart showing time doing head dipping. Statistical analysis shows a significant increase due to MS. Data are shown as Mean±SEM.
